# Exploring causal association between malnutrition, nutrients intake and inflammatory bowel disease: a Mendelian randomization analysis

**DOI:** 10.3389/fnut.2024.1406733

**Published:** 2024-08-14

**Authors:** Shi Wang, Jing Wang, Xinyao Meng, Shimin Yang, Luyao Wu, Ke Chen, Zejian Li, Jun Xiao, Xiaosi Yu, Xuyong Chen, Jiexiong Feng, Rui Gong

**Affiliations:** ^1^Department of Breast and Thyroid Surgery, Union Hospital, Tongji Medical College, Huazhong University of Science and Technology, Wuhan, China; ^2^Department of Pediatric Surgery, Tongji Hospital, Tongji Medical College, Huazhong University of Science and Technology, Wuhan, China; ^3^Tongji Medical College, Huazhong University of Science and Technology, Wuhan, China; ^4^Health Management Center, Union Hospital, Tongji Medical College, Huazhong University of Science and Technology, Wuhan, China

**Keywords:** malnutrition, nutrients intake, inflammatory bowel disease, Mendelian randomization, GWAS

## Abstract

**Background:**

Malnutrition has emerged as main side effects of inflammatory bowel disease (IBD) which might also affect the prognosis of IBD. However, whether these associations are causal remains unclear. We aimed to identify the causality of IBD on malnutrition and explore the causal relationship of malnutrition and nutrients intake on IBD by using Mendelian randomization (MR).

**Methods:**

Single nucleotide polymorphisms associated with IBD, malnutrition and nutrients intake were obtained from previous researches of genome-wide association studies (GWAS) (*p* < 0.00000005). MR analysis was conducted to evaluate the causality with different methods based on OR and their 95% CIs. Meanwhile, heterogeneity, pleiotropy and MR-PRESSO were used for instrumental variables evaluation.

**Results:**

The results of MR analysis revealed that IBD, both Crohn disease (CD) and ulcerative colitis (UC), could directly impact the incidence of malnutrition (*p*-value <0.01). CD is directly related to nutrients such as sugar, fat, VA, VC, VD and zinc, while UC is correlated with carbohydrate, fat, VB12, VC, VD, VE, iron, zinc and magnesium. However, our results suggested that malnutrition could not affect the risk of IBD directly (*p* > 0.05). Further analysis showed similar results that nutrients intake had no direct effect on IBD, neither CD or UC.

**Conclusion:**

Our results indicated that IBD increases the risk of malnutrition, however, malnutrition and nutrients intake might not directly affect the progression of IBD.

## Introduction

Inflammatory bowel disease (IBD) is a chronic, relapsing inflammatory gastrointestinal disorder comprising two primary forms, namely Crohn’s disease (CD) and ulcerative colitis (UC) (1). Clinical manifestations of IBD include abdominal pain, diarrhea, rectal bleeding, and weight loss (2). IBD is a multifactorial disease, and its pathogenesis remains unclear. Abnormal immune responses mediated within the intestinal microenvironment, microbiota, and genetic factors play pivotal roles in the development of IBD (3). Current understanding of the etiology of IBD dictates that management primarily revolves around anti-inflammatory and immunosuppressive strategies (4, 5).

Malnutrition was diagnosed based on Global Leadership Initiative on Malnutrition (GLIM) criteria (phenotypic criteria include unintentional weight loss, low BMI and low muscle mass) (6). IBD patients often exhibit reduced food intake, malabsorption, dysbiosis, and increased energy expenditure as a result of immune-inflammatory reactions, contributing to the development of malnutrition (7). More than 70% of hospitalized patients present symptoms of weight loss. Furthermore, poor nutritional status is closely linked to an unfavorable prognosis and inadequate response to treatment. Therefore, the assessment of nutritional status and the provision of appropriate nutritional support are vital components of the clinical treatment process (8, 9). While malnutrition in IBD patients may stem from various factors, and enhancing nutritional status is a crucial aspect of clinical care, the involvement of malnutrition in the pathogenesis of IBD remains uncertain (10, 11). Similarly, the potential impact of nutrient intake on the progression or remission of IBD necessitates further exploration.

Conventional observational epidemiological research methods are vulnerable to confounding factors and the possibility of reverse causation (12). Furthermore, prospective studies often entail substantial time and financial investments (13). Mendelian randomization (MR) represents an analytical technique utilized to deduce causal relationships between exposure factors and outcomes by leveraging genetic variations linked to the exposure factor (14, 15). Through the random assignment of alleles of these genetic variations, MR minimizes the influence of reverse causation and other confounding factors, thereby offering a significant reduction in the impact of such factors (16). This approach has garnered considerable attention in recent years.

In this study, we identified relevant genetic variations and performed a MR analysis to explore the reciprocal relationships between malnutrition and IBD (CD and UC). Furthermore, we conducted additional analyses to examine the links between essential nutritional components (carbohydrate, sugar, fat, protein, vitamin A, vitamin B12, vitamin C, vitamin D, vitamin E, calcium, iron, zinc and magnesium) and IBD (CD and UC). Our aim is that this study will potentially lay the groundwork for advancing the clinical management of IBD.

## Materials and methods

### Study design

**Figure 1:**
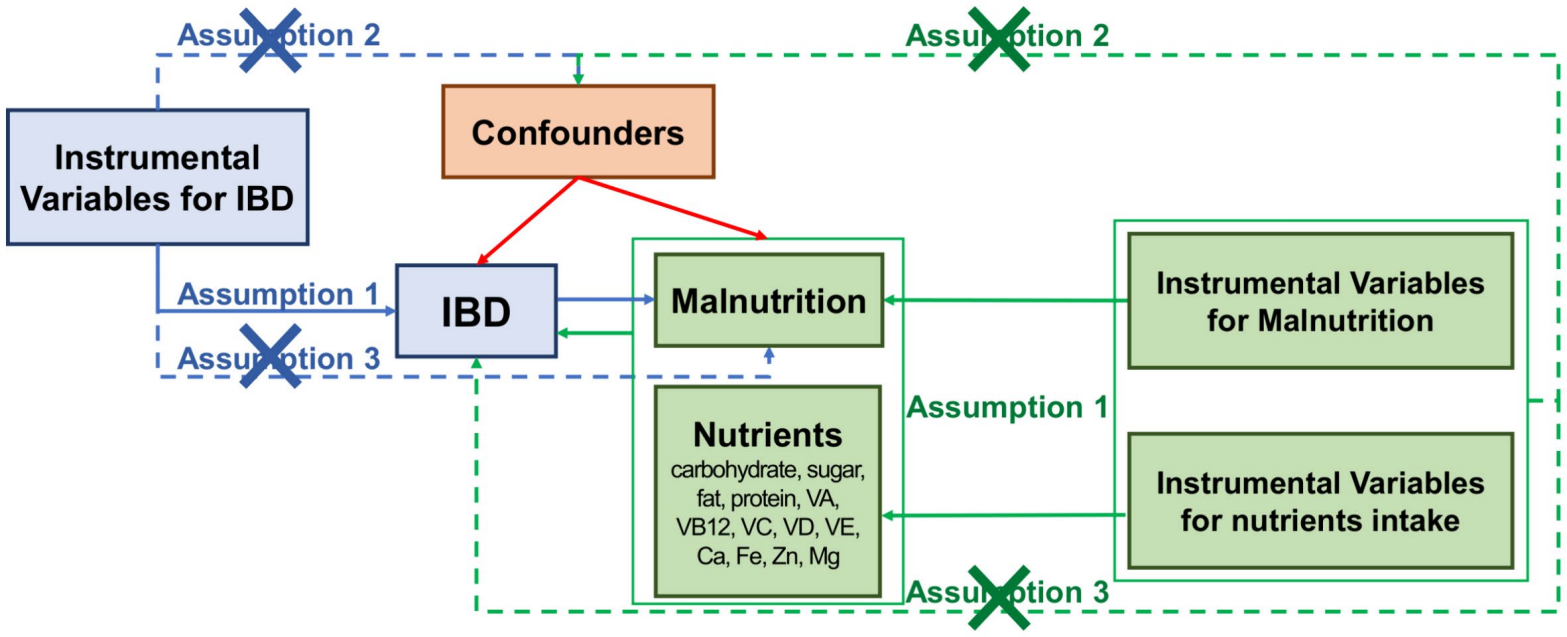
Overview of study design.

MR analysis assumes the distribution of gene variants in the population is random and these gene variants only influence the outcomes through the pathway of exposure factors and are not affected by other confounding factors. Figure 1 showed the flow chart of our study. Our MR study designed also followed the STROBE-MR statement and firstly examined the bidirectional associations between malnutrition and IBD (CD and UC). Meanwhile, the relationship between different nutrients (carbohydrate, sugar, fat, protein, vitamin A, vitamin B12, vitamin C, vitamin D, vitamin E, calcium, iron, zinc, magnesium) and IBD were also discussed.

### Data collection

The datasets our study involved were taken from publicly available GWAS (17, 18). SNPs associated with malnutrition and IBD were screened from GWAS meta-analysis. Single-nucleotide polymorphisms (SNPs) of nutrient elements were extracted from biobank (19). Detailed information of data used in our study is displayed in Table 1.

**Table 1:** Detailed information of data used in this study.

Phenotype	Sample size	Population	Consortium or cohort Study	Data source
IBD	199,947	European	FinnGen	K11_IBD_STRICT_PSC
Crohn disease	218,700	European	FinnGen	K11_CD_STRICT2
Ulcerative colitis	218,384	European	FinnGen	K11_UC_STRICT2
Malnutrition	377,277	European	FinnGen	E4_MALNUTRITION
Carbohydrate	64,979	European	UK Biobank	ukb-b-7244
Sugar	64,979	European	UK Biobank	ukb-b-17079
Fat	13,505	European	UK Biobank	met-c-936
Protein	210,947	European	UK Biobank	ukb-d-30860_irnt
Vitamin A	469,214	European	UK Biobank	ukb-b-9596
Vitamin B12	64,979	European	UK Biobank	ukb-b-19524
Vitamin C	64,979	European	UK Biobank	ukb-b-4690
Vitamin D	335,591	European	UK Biobank	ukb-a-462
Vitamin E	335,591	European	UK Biobank	ukb-a-463
Calcium	361,194	European	UK Biobank	ukb-d-30680_irnt
Iron	64,979	European	UK Biobank	ukb-b-20447
Zinc	64,979	European	UK Biobank	ukb-b-13891
Magnesium	64,979	European	UK Biobank	ukb-b-7372

### Instrument variants selection

Quality control techniques were conducted to screen instrument variants satisfying the first assumption. Meanwhile, independent SNPs were selected with *p*-value <5 × 10^−8^ and *r*^2^ < 0.001 to exclude linkage disequilibrium. All instrument variants are listed in Table 2.

**Table 2:** Statistic of sample size and instrumental variables.

Exposure		Outcome		Instrumental variables (nSNPs)
Trait	Sample size	Trait	Sample size	
IBD	199,947	Malnutrition	377,277	3,126
Crohn disease	218,700	Malnutrition	377,277	332
Ulcerative colitis	218,384	Malnutrition	377,277	2,893
Malnutrition	377,277	IBD	199,947	41
Malnutrition	377,277	Crohn disease	218,700	41
Malnutrition	377,277	Ulcerative colitis	218,384	41
Crohn disease	218,700	Carbohydrate	64,979	260
Crohn disease	218,700	Sugar	64,979	260
Crohn disease	218,700	Fat	13,505	233
Crohn disease	218,700	Protein	210,947	288
Crohn disease	218,700	Vitamin A	469,214	251
Crohn disease	218,700	Vitamin B12	64,979	260
Crohn disease	218,700	Vitamin C	64,979	216
Crohn disease	218,700	Vitamin D	335,591	261
Crohn disease	218,700	Vitamin E	335,591	261
Crohn disease	218,700	Calcium	361,194	288
Crohn disease	218,700	Iron	64,979	260
Crohn disease	218,700	Zinc	64,979	257
Crohn disease	218,700	Magnesium	64,979	260
Ulcerative colitis	218,384	Carbohydrate	64,979	1,924
Ulcerative colitis	218,384	Sugar	64,979	1,924
Ulcerative colitis	218,384	Fat	13,505	1,385
Ulcerative colitis	218,384	Protein	210,947	2,295
Ulcerative colitis	218,384	Vitamin A	469,214	1,871
Ulcerative colitis	218,384	Vitamin B12	64,979	1,924
Ulcerative colitis	218,384	Vitamin C	64,979	1,759
Ulcerative colitis	218,384	Vitamin D	335,591	1,887
Ulcerative colitis	218,384	Vitamin E	335,591	1,887
Ulcerative colitis	218,384	Calcium	361,194	2,295
Ulcerative colitis	218,384	Iron	64,979	1,924
Ulcerative colitis	218,384	Zinc	64,979	1,901
Ulcerative colitis	218,384	Magnesium	64,979	1,924
Carbohydrate	64,979	IBD	199,947	5
Fat	13,505	IBD	199,947	19
Protein	210,947	IBD	199,947	544

### Statistical analysis

The MR analysis was conducted by “TwoSampleMR” and “MRPRESSO” packages. The inverse variance weighted (IVW) method was used as the primary analysis method and maximum likelihood, MR-Egger and weighted median methods were complementary. The association of malnutrition with IBD was assessed by combining beta values and standard errors. Besides, MR heterogeneity and pleiotropy test were evaluated by MR-Egger and MR-pleiotropy.

## Results

### Causal effects of IBD on malnutrition

**Figure 2:**
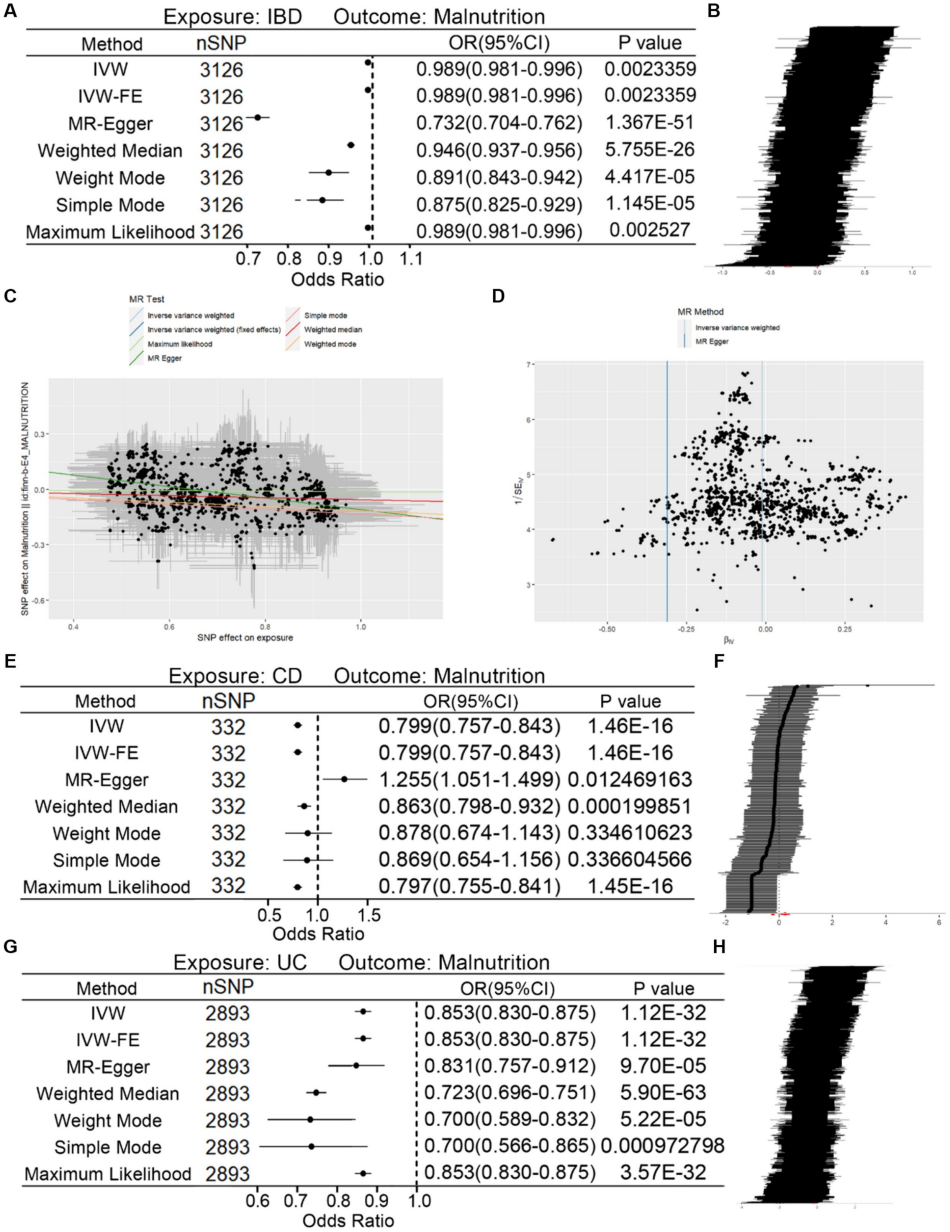
Associations of genetic predicted IBD instrumental variables with malnutrition. **(A)** Forest plot of causal relationship of IBD on malnutrition with different methods. **(B)** Sensitivity analysis of the association of IBD on malnutrition represented by SNPs. **(C,D)** Scatter plot and funnel plot of causality of IBD on malnutrition. **(E)** Forest plot of causal relationship of CD on malnutrition with different methods. **(F)** Sensitivity analysis of the association of CD on malnutrition represented by SNPs. **(G)** Forest plot of causal relationship of UC on malnutrition with different methods. **(H)** Sensitivity analysis of the association of UC on malnutrition represented by SNPs.

The results of MR analysis demonstrated the causal effect of IBD on malnutrition in all method which were presented in Figure 2A and these related SNPs were shown in Figure 2B. The scatter and funnel plot were also displayed in Figures 2C,D. Meanwhile, no heterogeneity (*p*-value >0.05) was found by Cochran’s *Q* test (Table 3). MR-Egger regression method detected directional pleiotropy (Intercept = 0.202, *p*-value = 1.026 × 10^−49^) (Table 3). Although MR-PRESSO analysis was not applicable for this data set, it would not have significant impacts on the conclusion that IBD is the risk factor of malnutrition. Moreover, the causal relationship of CD, UC on malnutrition were also revealed (Figures 2E–H). In addition, the relationship of CD, UC and malnutrition was also shown in scatter and funnel plots (Supplementary Figures S1A–D).

**Table 3:** Pleiotropy, heterogeneity and MR-PRESSO assessment.

Exposure	Outcome	Pleiotropy			Heterogeneity				MR-PRESSO global test
		Intercept	SE	*p*-value	Method	*Q*	df	*Q* value	*p*-value
IBD	Malnutrition	0.202	0.013	1.03 × 10^−49^	MR-Egger	1827.311	3,124	1	/
Crohn disease	Malnutrition	−0.131	0.025	2.91 × 10^−7^	MR-Egger	276.319	330	0.986	/
Ulcerative colitis	Malnutrition	0.009	0.009	0.329	MR-Egger	1645.892	2,468	1	1
Malnutrition	IBD	0.303	0.1	0.005	MR-Egger	25.433	39	0.954	0.85
Malnutrition	Crohn disease	−0.038	0.04	0.346	MR-Egger	29.337	39	0.869	0.563
Malnutrition	Ulcerative colitis	−0.028	0.023	0.242	MR-Egger	25.204	39	0.957	0.442
Crohn disease	Carbohydrate	0.005	0.002	0.008	MR-Egger	194.147	258	0.999	0.994
Crohn disease	Sugar	0.01	0.002	6.69 × 10^−6^	MR-Egger	211.727	258	0.984	0.839
Crohn disease	Fat	−0.027	0.004	9.23 × 10^−11^	MR-Egger	78.173	231	1	1
Crohn disease	Protein	−0.007	0.003	0.049	MR-Egger	4219.562	286	0	<0.001
Crohn disease	Vitamin A	−0.001	0	3.57 × 10^−15^	MR-Egger	277.838	249	1.01 × 10^−1^	<0.003
Crohn disease	Vitamin B12	−0.003	0.002	1.98 × 10^−1^	MR-Egger	330.061	258	0.002	<0.002
Crohn disease	Vitamin C	−0.005	0.001	2.17 × 10^−10^	MR-Egger	205.748	214	0.645	0.092
Crohn disease	Vitamin D	−0.001	0	6.17 × 10^−6^	MR-Egger	157.474	259	1	1
Crohn disease	Vitamin E	0	0	2.71 × 10^−1^	MR-Egger	112.26	259	1	1
Crohn disease	Calcium	−0.002	0.002	1.83 × 10^−1^	MR-Egger	937.497	286	2.66 × 10^−70^	<0.003
Crohn disease	Iron	−0.002	0.002	3.85 × 10^−1^	MR-Egger	50.788	258	1	1
Crohn disease	Zinc	0	0	1.20 × 10^−2^	MR-Egger	234.825	255	0.813	0.823
Crohn disease	Magnesium	0	0.002	9.29 × 10^−1^	MR-Egger	88.2	258	1	1
Ulcerative colitis	Carbohydrate	0.003	0	1.49 × 10^−7^	MR-Egger	1855.608	1,922	0.858	0.793
Ulcerative colitis	Sugar	0	0.001	7.46 × 10^−1^	MR-Egger	1346.554	1,922	1	1
Ulcerative colitis	Fat	0.009	0.992	7.13 × 10^−9^	MR-Egger	1874.44	1,383	1.66 × 10^−17^	<0.001
Ulcerative colitis	Protein	0.005	0.002	5.00 × 10^−3^	MR-Egger	102200.9	2,293	0	<4 × 10^−4^
Ulcerative colitis	Vitamin A	3.26 × 10^−5^	3.18 × 10^−5^	3.05 × 10^−1^	MR-Egger	1.94 × 10^3^	1.87 × 10^3^	1.13 × 10^−1^	0.12
Ulcerative colitis	Vitamin B12	0.004	0.001	2.29 × 10^−11^	MR-Egger	1105.621	1,922	1	1
Ulcerative colitis	Vitamin C	0.002	0	9.84 × 10^−29^	MR-Egger	1837.8	1,757	2.62 × 10^−2^	<0
Ulcerative colitis	Vitamin D	0.001	4.94 × 10^−5^	3.70 × 10^−38^	MR-Egger	1746.961	1,885	0.989	0.516
Ulcerative colitis	Vitamin E	0	4.99 × 10^−5^	1.09 × 10^−14^	MR-Egger	2507.384	1,885	1.68 × 10^−20^	<0
Ulcerative colitis	Calcium	0	0.00 × 10^0^	5.91 × 10^−1^	MR-Egger	8138.781	2,293	0	<4 × 10^−4^
Ulcerative colitis	Iron	0.01	1.00 × 10^−3^	6.65 × 10^−66^	MR-Egger	1750.615	1,922	9.98 × 10^−1^	0.022
Ulcerative colitis	Zinc	0.001	6.70 × 10^−5^	2.72 × 10^−41^	MR-Egger	4306.929	1,899	6.55 × 10^−188^	<0
Ulcerative colitis	Magnesium	0.009	1.00 × 10^−3^	1.53 × 10^−48^	MR-Egger	1887.033	1,922	7.11 × 10^−1^	0.003
Carbohydrate	IBD	0.208	3.15 × 10^−1^	5.56 × 10^−1^	MR-Egger	1	3	8.01 × 10^−1^	0.872
Fat	IBD	0.059	9.90 × 10^−2^	5.57 × 10^−1^	MR-Egger	15.871	17	5.33 × 10^−1^	0.56
Protein	IBD	0.037	1.60 × 10^−2^	1.90 × 10^−2^	MR-Egger	634.241	542	4.00 × 10^−3^	<0.001

### Causal effects of CD, UC on nutrient elements

**Figure 3:**
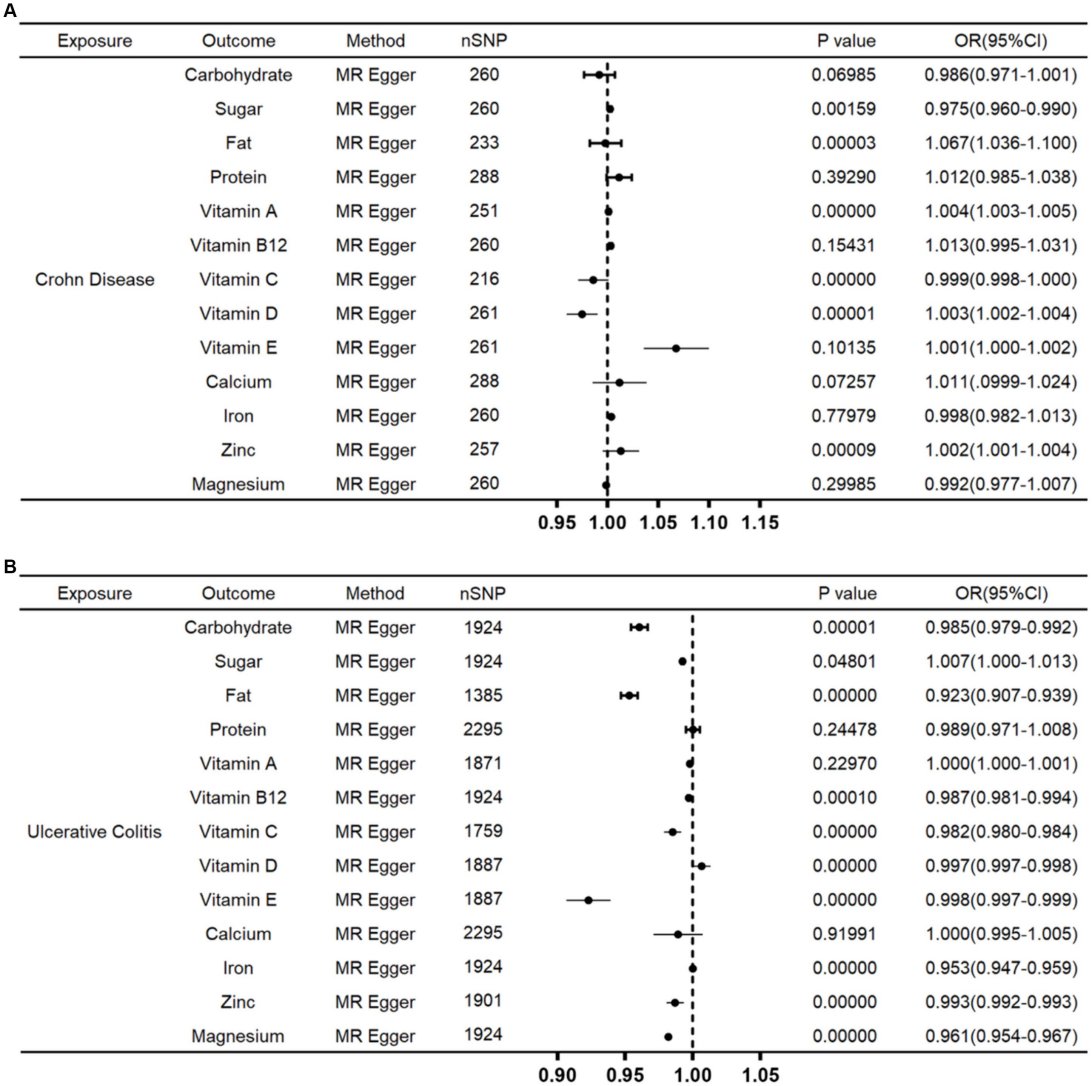
Associations of genetically instrumental variables predicting relationship of CD and UC on nutrient elements. **(A)** Forest plot of causal relationship of CD on nutrient elements. **(B)** Forest plot of causal relationship of UC on nutrient elements.

In addition, we also evaluated the causal effects of CD and UC on nutrient elements. We found that CD had a direct impact on the body’s nutrients level such as sugar, fat vitamin A, vitamin C, vitamin D and zinc (Figure 3A). Furthermore, UC could directly influence the level of carbohydrate, sugar, fat, vitamin B12, vitamin C, vitamin D, vitamin E, iron, zinc and magnesium (Figure 3B).

### Causal effects of malnutrition on IBD

**Figure 4:**
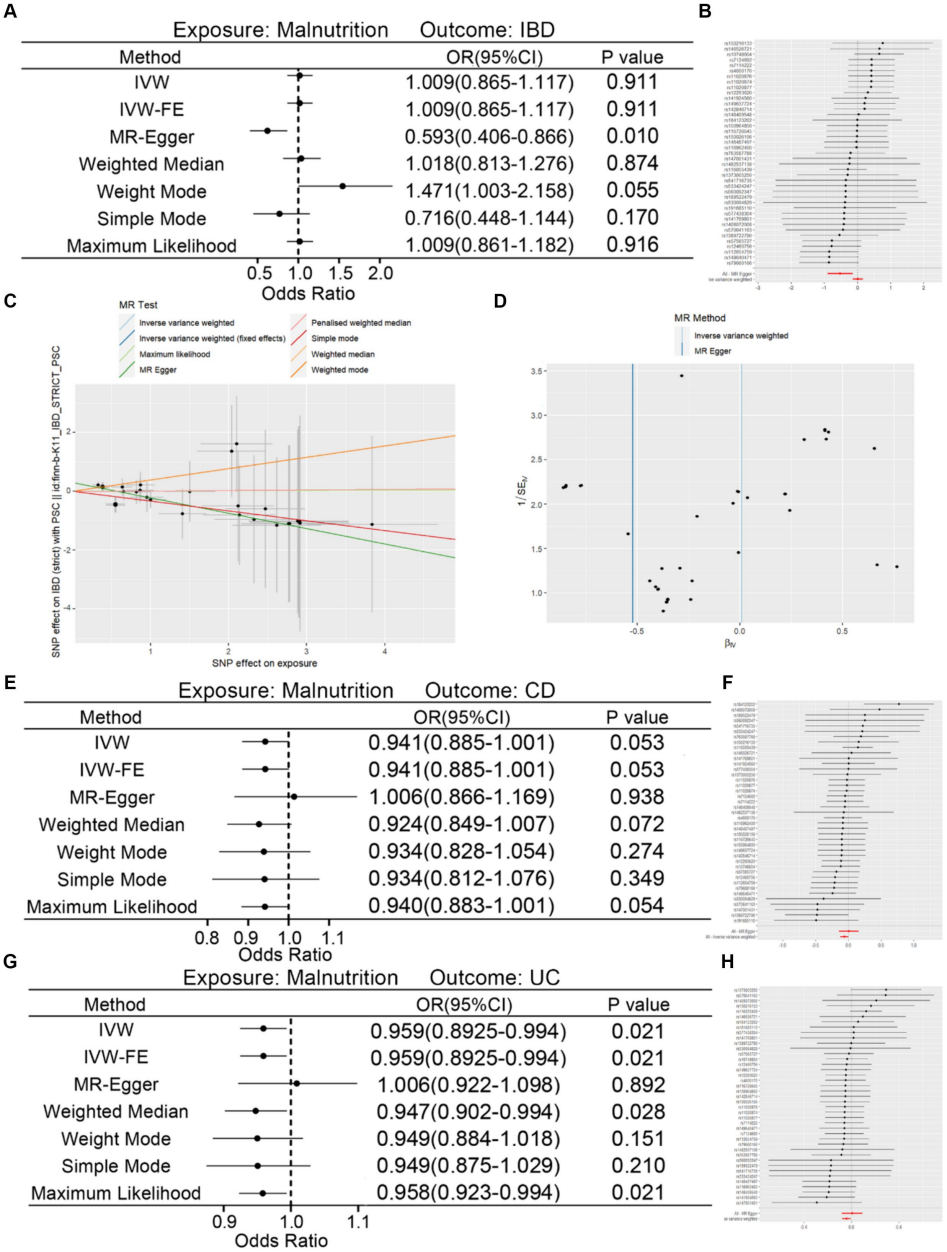
Associations of genetically instrumental variables predicting relationship of malnutrition on IBD. **(A)** Forest plot of causal relationship of malnutrition on IBD with different methods. **(B)** Sensitivity analysis of the association of malnutrition on IBD represented by SNPs. **(C,D)** Scatter plot and funnel plot of causality of malnutrition on IBD. **(E)** Forest plot of causal relationship of malnutrition on CD with different methods. **(F)** Sensitivity analysis of the association of malnutrition on CD represented by SNPs. **(G)** Forest plot of causal relationship of malnutrition on UC with different methods. **(H)** Sensitivity analysis of the association of malnutrition on UC represented by SNPs.

After synthesizing the results of various methods, we indicated that there was no causal relationship for malnutrition to IBD (Figures 4A,B). The scatter and funnel plot were also displayed in Figures 4C,D. Besides, no heterogeneity (*p*-value >0.05) was found but directional pleiotropy (Intercept = 0.303, *p*-value = 0.005) was detected (Table 3). After eliminating outliers, results of MR-PRESSO analysis showed no directional pleiotropy remained (Table 3). After that, we also analyzed the causal effects of malnutrition on CD and UC. Similarly, malnutrition could not directly lead to CD or UC (Figures 4E–H). The scatter and funnel plots were shown in Supplementary Figures S2A–D.

### Causal effects of nutrients intake on IBD

**Figure 5:**
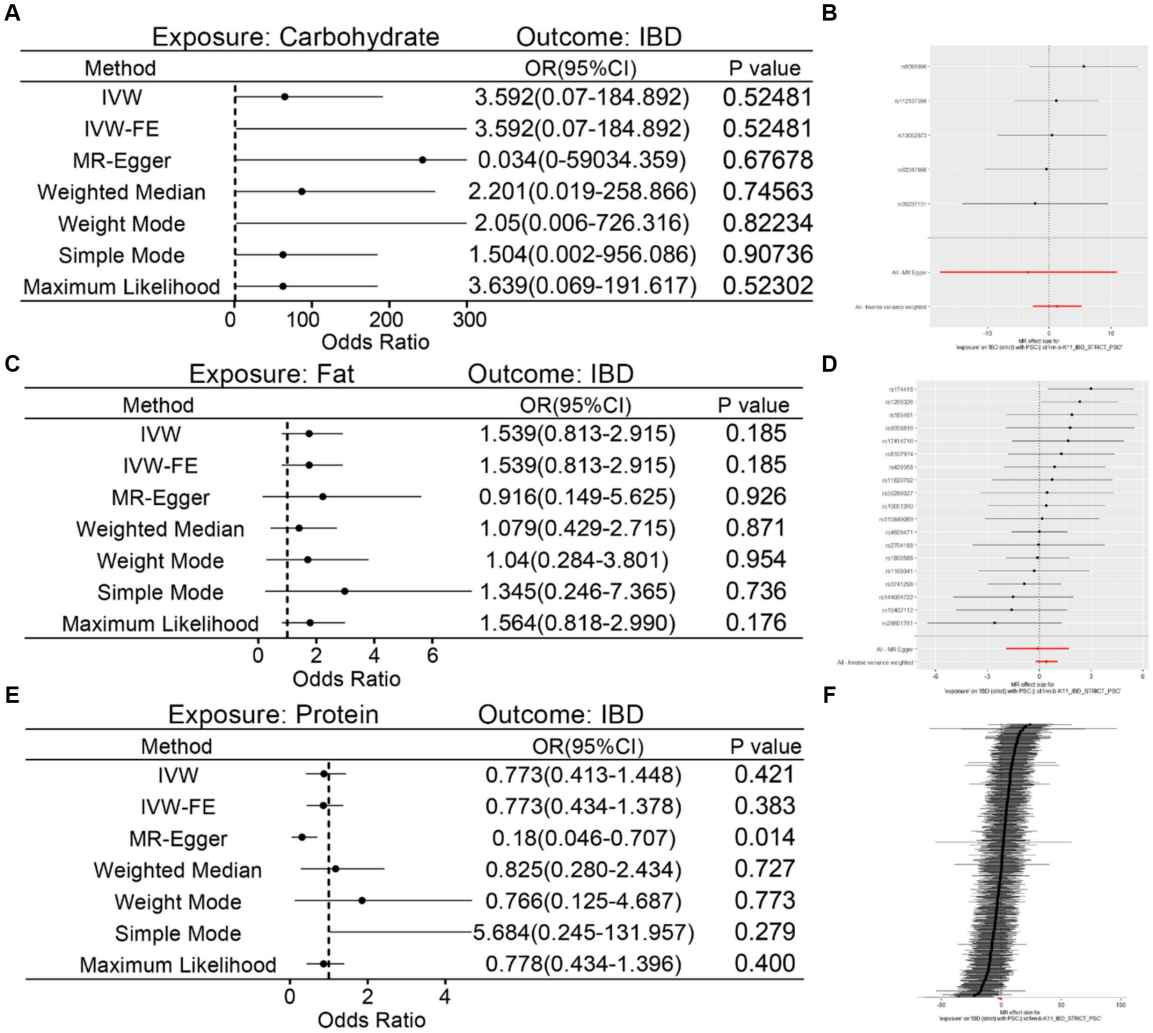
MR analysis of causal relationship of nutrients intake on IBD. **(A,B)** Detailed causality of carbohydrate intake on IBD. **(C,D)** Detailed causality of fat intake on IBD. **(E,F)** Detailed causality of protein intake on IBD.

Finally, the causal relationship of major nutrients intake (carbohydrate, fat, protein) on IBD was also evaluated and the results of MR analysis revealed that these three nutrients intake were not associated with IBD (Figures 5A,C,E). The Cochran’s *Q* test showed that heterogeneity only existed between protein and IBD (*p*-value <0.05) and the other two showed no heterogeneity with IBD (Table 3). Similarly, directional pleiotropy existed only between protein and IBD (Table 3). Through outliers removement and MR-PRESSO analysis, the pleiotropy of protein on IBD has not been eliminated (Table 3). Furthermore, risk of SNPs among different instrumental variables were shown in Figures 5B,D,F. Finally, the causal relationship, scatter and funnel plots of nutrients intake on IBD were also shown (Supplementary Figures S3A–F).

## Discussion

This study firstly investigated the causal relationship between malnutrition, nutrients intake and IBD. Through MR analysis, we ultimately concluded that IBD is likely to increase the risk of malnutrition, which was consistent with the conclusions of previous clinical researches. Meanwhile, we failed to find compelling evidence that genetically predicted the causal relationship of malnutrition and nutrients intake to IBD.

The correlation between malnutrition and the risk of IBD has been reported previously by epidemiological observations (20). Due to the long-term course of disease, IBD patients usually experience loss of appetite and reduced food intake (21). Besides, glucocorticoids and sulfasalazine treatment could also result in malabsorption of nutrients, nausea and anemia (22). Meanwhile, diarrhea and vomiting further reduce nutrients intake. In addition, prolonged inflammatory state and imbalance of intestinal flora homeostasis seriously affect intestinal digestion and absorption. After that, long-term state of unhealthy and abnormal immune response aggravates energy consumption. Finally, prolonged restrictive diet management would also lead to a significant reduction of nutrients intake. Combined with these reasons mentioned above, there are sufficient grounds to reach the conclusion that IBD could increase the risk of malnutrition (7).

Our MR analysis demonstrated the same causal relationship of IBD on malnutrition. The results revealed several SNPs directly associated with IBD and malnutrition, which supported that multiple pathways were involved in the pathogenesis of IBD induced malnutrition. Besides, both CD and UC could result in malnutrition. These results are consistent with the results of clinical study.

Interestingly, several nutrient elements were found to be directly correlated with IBD, such as fat, vitamin C, vitamin D and minerals (iron, zinc). Previous research has indicated that fat distribution of viscera was valuable for subtype distinguish of IBD (23). Long chain fatty acid intake might potentially increase the risk of colitis, which were not found in our research (24). Besides, clinical study also found a widespread decrease of VC in blood samples from IBD patients, which was consistent with results of MR analysis (25). Another research has elaborated the application value of Vitamin Din the treatment of autoimmune diseases, especially IBD (26).

In addition, nutritional support therapy is receiving increasing attention in clinical practice of IBD (27). Recently, many researches have discussed the role of nutritional support in the treatment of IBD and it has been included in relevant diagnosis and treatment guidelines (11, 28, 29). Precise dietary management were found to improve disease activity and prolong relapse interval to some extent, however, the specific clinical value and mechanism of diet management in the treatment of IBD remains unclear (30, 31).

Due to the limitation of observational research methods and the difficulty of clinical implementation of prospective studies, our study used SNPs specifically related to exposure or outcome as instrumental variables to evaluate the causal relationship of malnutrition and nutrients intake on the risk of IBD. To our surprise, the results were inconsistent with clinical experience: (1) malnutrition could not increase the risk of IBD; (2) intake of carbohydrate, fat and protein had no effect on the occurrence of IBD. We speculated that nutritional status and nutrients intake might not directly affect the progression of IBD. Firstly, nutritional support therapy is mostly decided based on the nutritional status of IBD patients. Clinical nutritional support is expected to restore the immune capacity of IBD patients and supplement the nutritional loss in the course of disease to a certain extent. Malnutrition may not have a direct effect on IBD. Similarly, the effects of nutrients intake on IBD might also be indirect. Due to the characteristics of MR analysis, mediated SNPs may have been filtered out at the beginning of our analysis, which requires further researches. Nutritional status might affect IBD through improving the immune level, regulating the homeostasis of intestinal flora and protecting the normal function of intestinal tract. Certainly, the mechanism that SNPs that mediated directly with malnutrition identified in this research remains further exploration.

## Conclusion

The result of our MR analysis strongly supports the clinical findings that IBD (both CD and UC) is causally related to risk of malnutrition but there is insufficient evidence to suggest that malnutrition and nutrients intake have direct effects on IBD.

## Data availability statement

The original contributions presented in the study are included in the article/Supplementary material, further inquiries can be directed to the corresponding authors.

## Author contributions

SW: Formal analysis, Methodology, Software, Funding acquisition, Writing – original draft. JW: Conceptualization, Formal analysis, Methodology, Software, Writing – original draft. XM: Conceptualization, Formal analysis, Methodology, Software, Writing – original draft. SY: Data curation, Visualization, Writing – review & editing. LW: Data curation, Writing – review & editing. KC: Data curation, Writing – review & editing. ZL: Data curation, Writing – review & editing. JX: Data curation, Writing – review & editing. XY: Data curation, Writing – review & editing. XC: Data curation, Writing – review & editing. JF: Funding acquisition, Project administration, Resources, Supervision, Validation, Writing – review & editing. RG: Funding acquisition, Project administration, Resources, Supervision, Validation, Writing – review & editing.
