# Supplementary figures and images for: Exploring causal association between malnutrition, nutrients intake and inflammatory bowel disease: a Mendelian randomization analysis
Source: Front Nutr. 2024 Aug 14;11:1406733. doi: 10.3389/fnut.2024.1406733 (PMC11349745; doi:10.3389/fnut.2024.1406733)

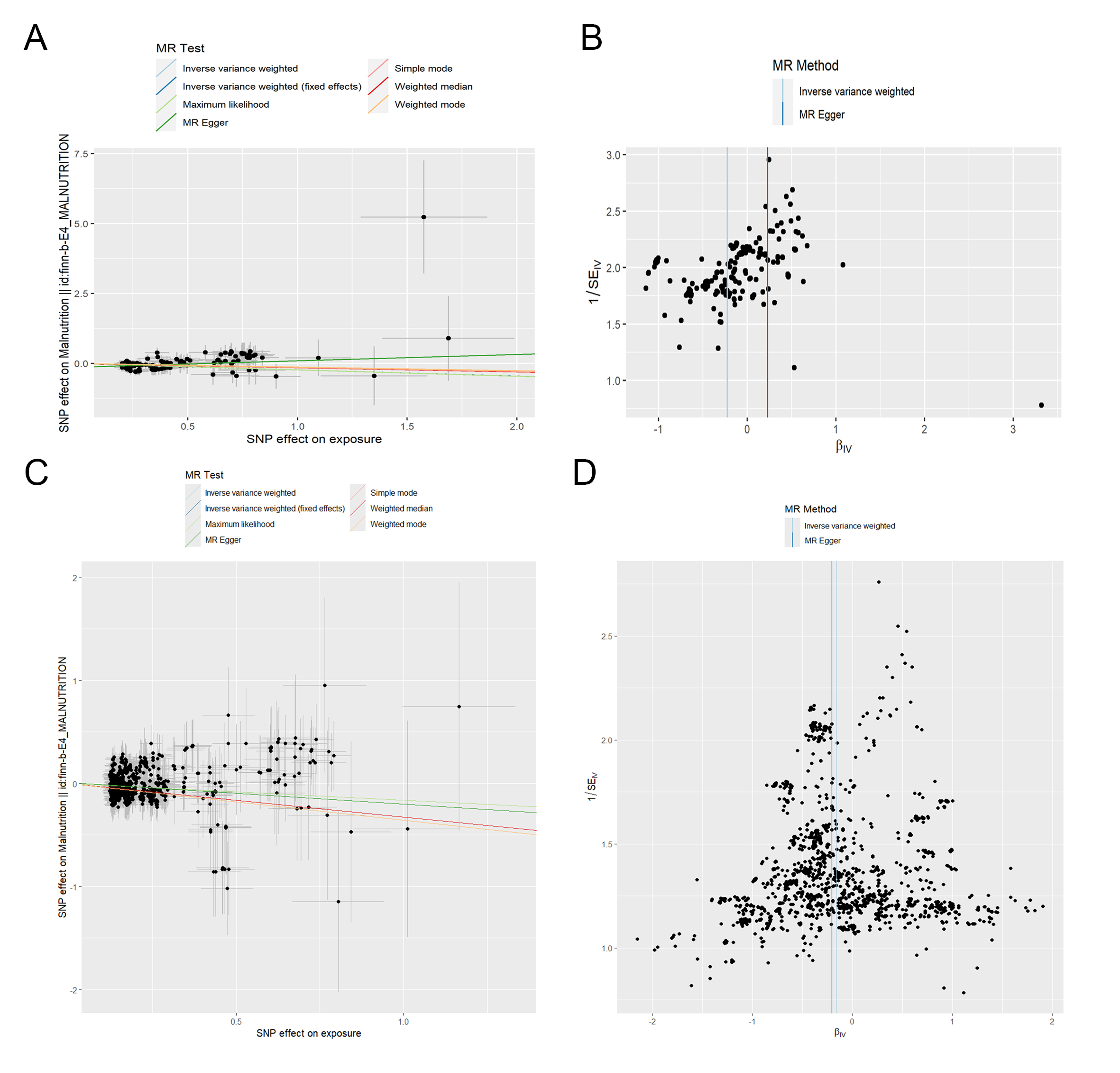

Supplement: Supplementary file 1 [file Image_1.JPEG]

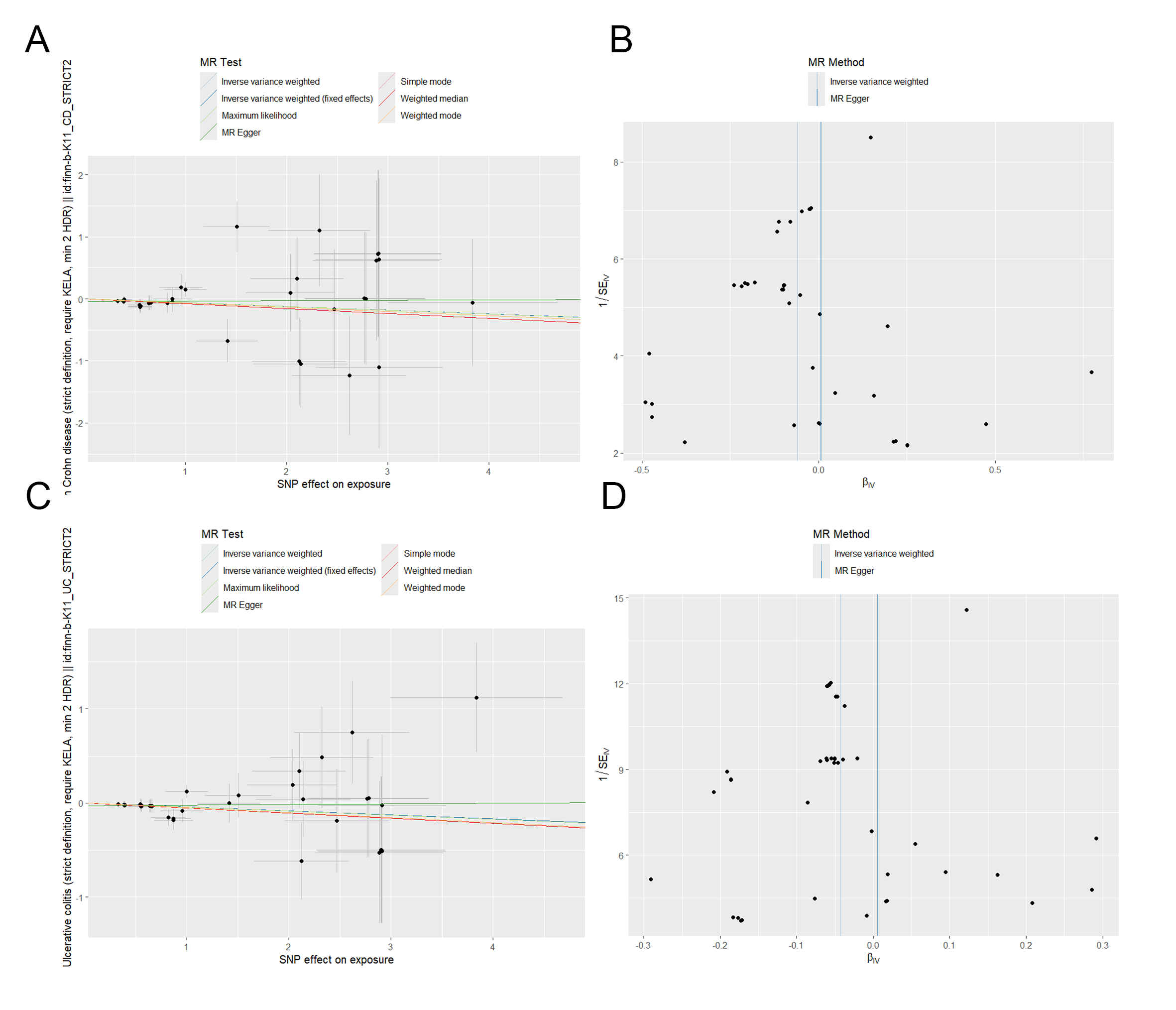

Supplement: Supplementary file 2 [file Image_2.JPEG]

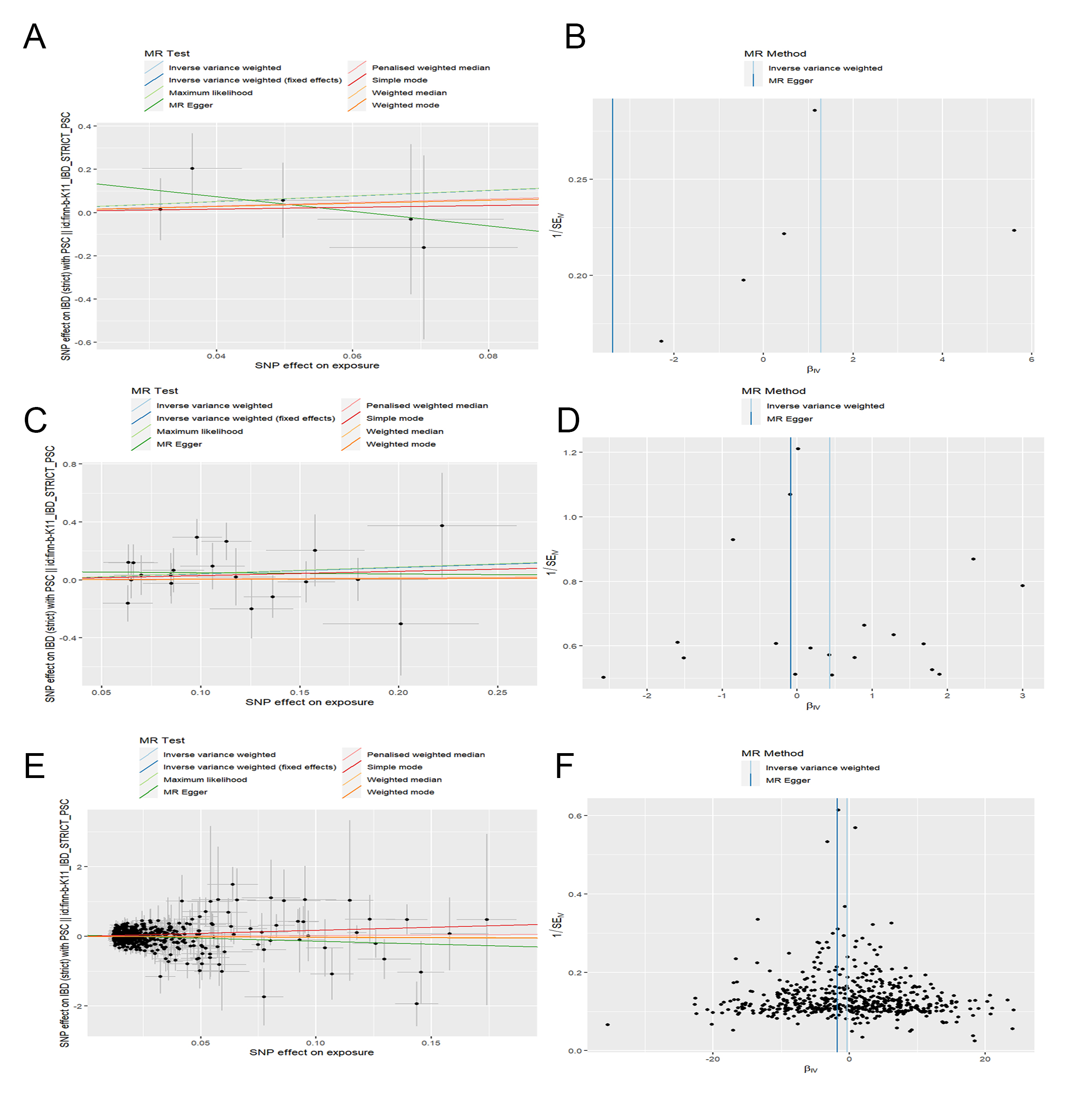

Supplement: Supplementary file 3 [file Image_3.JPEG]
